# Relationship Between Depression, Anxiety, Stress, and Weight Self-Stigma Among Youths in Saudi Arabia: A Nationwide Study

**DOI:** 10.7759/cureus.54125

**Published:** 2024-02-13

**Authors:** Abdullah A Ali, Abdulrahman A Aqeel, Mohammed O Shami, Bayan H Khodari, Ahmad Y Alqassim, Abdulrahman M Alessa, Feras I Alhazm, Yahya Alhasan Alhazmi, Abdulrrahman Y Sumayli, Fatimah H Arishi, Mohammed A Muaddi, Abdullah A Alharbi, Hatim A Almaghrabi, Abdulaziz H Alhazmi

**Affiliations:** 1 Family and Community Medicine Department, Faculty of Medicine, Jazan University, Jazan, SAU; 2 Family Medicine Department, Ministry of Health, Jazan, SAU; 3 Department of Medicine and Surgery, College of Medicine, Umm Al-Qura University, Makkah, SAU; 4 Pathology Department, Faculty of Medicine, Jazan University, Jazan, SAU

**Keywords:** saudi arabia, dass-21, stress, anxiety, depression, adolescent medicine, public health, youth, weight self-stigma

## Abstract

Background and objectives

Many research studies show that self-stigma related to weight can exacerbate mental health issues. There is also evidence suggesting that depression, anxiety, and stress could be predictors of weight stigma. However, these connections have not been thoroughly investigated among young people in Saudi Arabia, where there is a high prevalence of obesity. This study aimed to explore the relationships between depression, anxiety, stress, and weight self-stigma in Saudi Arabian adolescents and young adults.

Materials and methods

This cross-sectional study was conducted between January and March 2022 and utilized online surveys, including the Weight Self-Stigma Questionnaire (WSSQ) and Depression, Anxiety, and Stress Scale 21 (DASS-21). We conducted descriptive analysis, independent samples t-tests, analysis of variance (ANOVA), and linear regression for the statistical analysis using SPSS version 25 (IBM Corp., Armonk, NY).

Results

A total of 1624 participants were enrolled in this survey. Most participants (889, 54.7%) were females. The mean age was 20.73 years (SD: 2.63). Males were more likely to report self-stigma compared to females (p = 0.018). Weight self-stigma demonstrated significant positive associations with body mass index (BMI) (p = 0.0001) and depression, anxiety, and stress (p = 0.0001) scores. Further analysis revealed weight self-stigma was positively correlated with psychological distress levels in the study population (p = 0.0001). The results demonstrated a statistically significant difference between different regions of Saudi Arabia with weight self-stigma being more prevalent in the Al-Baha region and least prevalent in the Al-Madinah region.

Conclusions

The results of this study indicate robust positive correlations between weight self-stigma scores and depression, anxiety, and stress scale scores among adolescents and young adults in Saudi Arabia. Further epidemiological and clinical studies on the national level are warranted.

## Introduction

The World Health Organization (WHO) considers good mental and psychological health for optimal well-being and wellness. They are constantly focusing on preventing and managing mental health disorders through various programs for a better quality of life [1]. It is noteworthy that mental health disorders are among the leading causes of disabilities and significantly impair both physical and mental performance. Moreover, mental disorders pose a major public health issue globally due to their high prevalence, complex causal factors, disease progression, and difficulties with management [2,3]. Stress, depression, and anxiety are among the most common mental disorders that adversely impact psychological well-being and quality of life [4,5]. These conditions can also lead to various clinical manifestations affecting emotional health [6,7].

There are two types of stigmatizations that are highly associated with mental illnesses. These are public stigma and self-stigma. Self-stigmatization usually develops after societal changes, awareness of public stigmatization, and acceptance of general assumptions and self-limiting beliefs [8]. Notably, among other stigmas, weight stigma develops with discrimination or discriminatory behavior of the public, negative attitudes toward obese or overweight individuals, low self-esteem, and feelings of shame and humiliation [9-11]. The weight stigma among adults develops adverse biopsychosocial health outcomes and academic consequences. Medical research studies reported serious consequences of weight stigma among adults, such as anxiety, depression, and suicidal ideation [12-14]. Prior research has found that women with a higher body mass index (BMI) are more susceptible to stress compared to women with a lower BMI [15-19]. Plus, the weight stigma is considered a known risk factor for mental illnesses [14,20,21]. Some research studies showed that overweight adolescents are often bullied by their peers, which results in depression, stress, and sleep disturbances [18].

Studies in Asian countries have examined links between weight stigma, disordered eating attitudes and behaviors, and mental health outcomes. For example, a study of medical students in Thailand found that 15.9% had abnormal eating attitudes and behaviors associated with higher BMIs. These were connected to nutritional knowledge and long-term well-being [22]. Given concerning rates of obesity and mental health issues in Saudi youth, it is critical to study the relationships between weight stigma and psychological problems like depression, anxiety, and stress in this population.

In Saudi Arabia, overweight and obesity are highly prevalent among females according to the WHO [23]. A study reported that 38%, 49%, and 35% of boys aged 15-18 years suffer from depression, anxiety, and stress, respectively [24]. However, there is insufficient research data on mental health, psychological illnesses, and weight stigma among adolescents in Saudi Arabia. Given the concerning rates of obesity and mental health issues in Saudi youth, it is critical to study the relationships between weight stigma and psychological problems like depression, anxiety, and stress in this population.

The purpose of this nationwide study is to investigate the association between depression, anxiety, stress, and weight self-stigma among youth in Saudi Arabia. We aim to elucidate the role of weight bias in driving adverse mental health outcomes in overweight/obese Saudi adolescents. Understanding these relationships can ultimately inform clinical and public health efforts to reduce stigma and promote well-being.

## Materials and methods

Study design and participants

A cross-sectional, observational study was conducted between January and March 2022. The sample size was calculated using the Raosoft sample size calculator (Raosoft Inc., Seattle, WA) (http://www.raosoft.com/samplesize.html) based on the total population (35,013,414) according to the last report from the General Authority for Statistics in Saudi Arabia [25]. The minimum sample size was calculated to be 377 with a 95% confidence interval and a 5% margin of error. However, to reduce sampling bias in our method, as this study was based on an online questionnaire, we increased the sample size to include 1624 participants. Data were collected using an online survey that was actively distributed through e-mails and social media websites (Facebook, Twitter, and WhatsApp). The study included Arabic-speaking young adults aged 15 to 24 years who were living in Saudi Arabia.

Study tool

Participants first answered questions regarding sociodemographic factors like gender, age, employment, marital status, education level, income, and residence.

The Weight Self-Stigma Questionnaire (WSSQ) measured self-devaluation and fear of enacted stigma related to weight in two subscales. Prior research supports the reliability and validity of the Arabic WSSQ for assessing weight self-stigma cross-culturally [25,26].

The Depression, Anxiety, and Stress Scale 21 (DASS-21) is a validated 21-item Arabic self-report survey assessing depression, anxiety, and stress symptoms over the past week. Each subscale has seven items rated on a 0-3 scale. Subscale scores range from 0 to 42 with cutoff points of 20, 14, and 25 indicating severe levels of depression, anxiety, and stress, respectively. The DASS-21 has demonstrated strong psychometric properties in past studies with Cronbach's alpha values of 0.94 for depression, 0.87 for anxiety, and 0.91 for stress [27].

Data analysis

After data collection, data were verified manually and then coded into an Excel sheet (Microsoft Corporation, Redmond, WA). Statistical analysis was run using SPSS version 25 (IBM Corp., Armonk, NY). Data were analyzed using descriptive and comparative statistics. Descriptive statistics were calculated for study variables, e.g., frequency and percentage for qualitative variables and mean and standard deviation for quantitative variables. Associations between variables were calculated using independent samples t-test or one-way analysis of variance (ANOVA) as appropriate. Post-hoc Tukey’s honestly significant difference (HSD) was performed to examine significant associations in detail. A p-value of <0.05 was considered statistically significant. The specific statistical tests used included t-tests to compare weight stigma and mental health scores between males and females, ANOVA to analyze differences in weight stigma and mental health by BMI category, and regression models to assess the relationships between weight stigma, BMI, and psychological distress. The post-hoc Tukey test was used after ANOVA to make pairwise comparisons between BMI groups.

Ethical approval

The study received ethical approval from the Jazan Health Ethics Committee (reference #: 2157). For participants under 18 years old, parental consent was obtained prior to participation. The research complied with institutional and national ethical standards, the 1964 Helsinki Declaration, and its later amendments.

## Results

Demographic characteristics of the sample

A total of 1624 participants were enrolled in this survey; 889 (54.7%) were females. The mean age was 20.73 years (SD: 2.63). The demographic characteristics of the sample are shown in Table 1. As per education, 892 (54.9%) had at least a bachelor’s degree. Monthly income was almost evenly distributed among participants, with 488 (30.0%) reporting an income of more than 15,000 Saudi riyals. Regarding BMI, 258 (15.9%) were classified as underweight, 791 (48.7%) as normal, 325 (20.0%) as overweight, and 214 (13.2%) as obese (Table 1). The study included participants from different regions of Saudi Arabia (Table 2).

**Table 1:** Weight-self stigma and psychological distress in different demographic variables M, mean; SD, standard deviation; BMI, body mass index; SAR, Saudi riyals. Independent samples t-test or one-way analysis of variance (ANOVA) was used as appropriate. Post-hoc Tukey’s honestly significant difference test is as follows: ^†^ Percentages do not add up to 100 because of missing values, N = 36 (2.2%). ^a^ p < 0.01 vs. underweight. ^b^ p < 0.01 vs. normal weight. ^c^ p < 0.01 vs. overweight. ^d^ p < 0.01 vs. obese. ^*^ p < 0.05. ^**^ p < 0.01.

Variables/categories	Self-devaluation				Fear of enacted stigma		
	Total, N (%)	M	SD	F or t	M	SD	F or t
BMI class^†^							
Underweight (<18.5 kg/m^2^)	258 (15.9)	15.90^b,c,d^	6.16	67.611^**^	14.97^cd^	6.56	30.540^**^
Normal weight (18.5 to <25 kg/m^2^)	791 (48.7)	18.18^a,c,d^	6.14		16.08^cd^	6.71	
Overweight (25.0 to <30 Kg/m^2^)	325 (20.0)	20.74^a,b,d^	4.81		17.52^abd^	5.89	
Obese (≥30 kg/m^2^)	214 (13.2)	22.58^a,b,c^	5.05		20.10^abc^	5.88	
Sex							
Male	735 (45.3)	19.13	6.17	1.276	17.17	6.68	2.366^*^
Female	889 (54.7)	18.74	6.04		16.40	6.49	
Education							
Middle school	60 (3.7)	18.93	5.22	0.011	17.57	6.35	0.490
High school	672 (41.4)	18.95	6.36		16.69	6.87	
Bachelor	892 (54.9)	18.90	5.95		16.74	6.39	
Monthly income (SAR)							
0−4,999	394 (24.3)	19.01	5.99	0.473	17.26	6.63	2.710^*^
5,000−9,999	349 (21.5)	18.91	6.00		16.90	6.39	
10,000−15,000	393 (24.2)	19.14	6.04		16.94	6.47	
>15,000	488 (30.0)	18.67	6.30		16.07	6.75	

**Table 2:** Weight-self stigma and psychological distress in different regions of Saudi Arabia Independent samples t-test or one-way analysis of variance (ANOVA) was used as appropriate. Post-hoc Tukey’s honestly significant difference test is as follows: ^*^ p < 0.05. ^**^ p < 0.01.

Variables/categories	Total, N (%)	Self-devaluation			Fear of enacted stigma		
		M	SD	F or t	M	SD	F or t
Makkah	296 (18.2)	18.81	5.62	5.411^**^	16.50	6.12	8.962^**^
Madinah	203 (12.5)	17.11	6.58		14.22	6.16	
Jazan	183 (11.3)	19.37	6.08		16.46	6.91	
Qassim	103 (6.3)	19.56	5.84		16.75	6.33	
Riyadh	136 (8.4)	19.42	6.33		17.70	6.54	
Northern borders	88 (5.4)	19.42	5.74		18.38	5.87	
Tabuk	109 (6.7)	19.80	5.55		18.32	6.06	
Jouf	111 (6.8)	17.70	6.93		16.32	7.24	
Hail	96 (5.9)	17.45	6.50		15.18	6.49	
Najran	57 (3.5)	19.63	5.41		18.37	6.58	
Aseer	70 (4.3)	17.94	5.99		15.66	6.23	
Eastern	113 (7.0)	20.02	5.79		17.09	7.12	
Baha	59 (3.6)	22.71	4.01		22.49	4.69	

Weight self-stigma

The WSSQ was divided into two subscales: self-devaluation and enacted self-stigma. A series of one-way ANOVA (with post hoc Tukey's HSD test) tests and independent sample t-tests were conducted to explore the association between WSSQ subscales and different independent variables.

Self-devaluation

The respondents' mean scores on the self-devaluation subscale of WSSQ are summarized in Tables 1-3. Pearson's correlation coefficient showed that age (r(1624) = 0.097, p < 0.001) differed significantly according to the mean score of self-devaluation (Table 1). BMI (F(3, 1584) = 67.611, p < 0.001) was observed to be significantly associated with self-devaluation scores. Post hoc analyses suggested that the mean of self-devaluation exhibited a linear correlation with BMI (Table 1). The region of residence (F(12, 1611) = 5.411, p < 0.001) showed a significant association with the mean score of self-devaluation (Table 2). Respondents from the Al-Baha region had the highest self-devaluation scores (M = 22.71, SD = 4.01), and those from Al-Madinah had the lowest scores (M = 17.11, SD = 6.85). Depression (t(1622) = -3.747, p < 0.001), anxiety (t(1622) = -5.508, p < 0.001), and stress (t(1622) = -3.883, p < 0.001) were strongly associated with self-devaluation. Self-devaluation was more likely to be reported by participants with severe forms of psychological problems (Table 3).

**Table 3:** Weight-self stigma and psychological distress in different psychological problems Independent samples t-test or one-way analysis of variance (ANOVA) was used as appropriate. Post-hoc Tukey’s honestly significant difference test is as follows: * p < 0.05. ** p < 0.01.

Variable	Variable categories	Total, N (%)	Self-devaluation			Fear of enacted stigma		
			M	SD	F or t	M	SD	F or t
Depression	Not severe	1242 (76.5)	18.62	6.19	-3.747^**^	16.37	6.65	-4.308^**^
	Severe	382 (23.5)	19.90	5.69		17.97	6.25	-4.308^**^
Anxiety	Not severe	1071 (65.9)	18.35	6.33	-5.508^**^	15.86	6.72	-7.956^**^
	Severe	553 (34.1)	20.02	5.46		18.46	5.97	-7.956^**^
Stress	Not severe	1371 (84.4)	18.68	6.13	-3.883^**^	16.46	6.58	-4.117^**^
	Severe	253 (15.6)	20.23	5.76		18.28	6.44	-4.117^**^

Fear of enacted stigma

The respondents' mean scores on the enacted self-stigma subscale of WSSQ are summarized in Tables 1-3. Pearson's correlation coefficient showed that age (r(1624) = 0.075, p < 0.001) differed significantly according to the mean score of enacted self-stigma (Table 1). Males were more likely to report self-stigma compared to females (t(1622) = 2.366, p-value = 0.018). Respondents who reported a monthly income of more than 15,000 SAR were less likely to have self-stigma (F(3, 1620) = 2.710, p-value = 0.044). BMI (F(3, 1584) = 30.540, p < 0.001) was observed to be significantly associated with enacted self-stigma score. Post hoc analyses suggested that the mean of enacted self-stigma exhibited a linear correlation with BMI (Table 1). The region of residence (F(12, 1611) = 8.962, p < 0.001) showed a significant association with the mean score of enacted self-stigma (Table 2). Respondents from the Al-Baha region had the highest enacted self-stigma scores (M = 22.49, SD = 4.69), and those from Al-Madinah had the lowest scores (M = 14.22, SD = 6.16). Depression (t(1622) = -4.308, p < 0.001), anxiety (t(1622) = -7.956, p < 0.001), and stress (t(1622) -4.117, p < 0.001) were strongly associated with enacted self-stigma. Enacted self-stigma was more likely to be reported by participants with severe forms of psychological problems (Table 3).

Weight-self stigma and psychological distress

**Figure 1:**
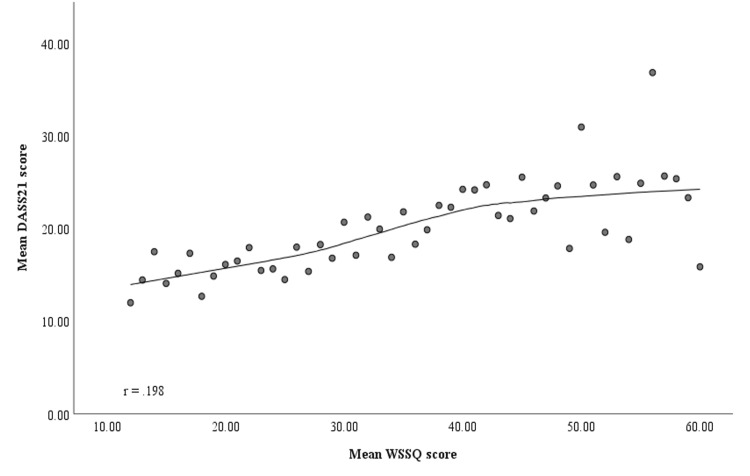
Scatterplot depicting the positive correlation between weight self-stigma scores and psychological distress levels WSSQ, Weight Self-Stigma Questionnaire; DASS-21, Depression, Anxiety, and Stress Scale 21.

Analysis of the correlation between subjects' mean weight self-stigma and DASS-21 scores revealed a statistically significant positive linear relationship (r = 0.198, p < 0.001). This indicates that higher weight self-stigma was associated with greater psychological distress in the study population (Figure 1).

**Figure 2:**
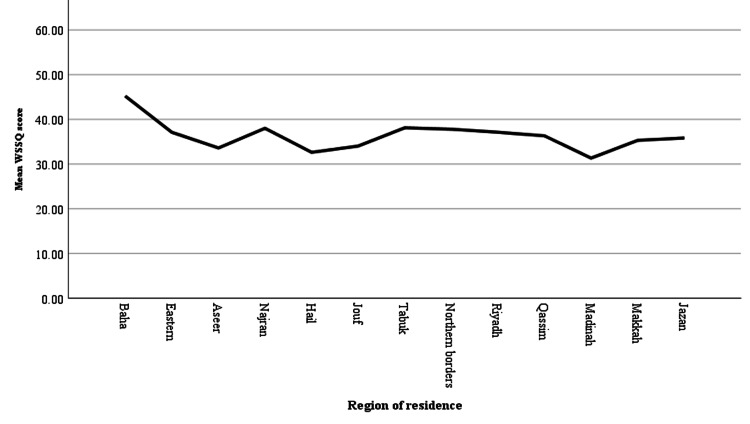
Line graph of the correlation between the Weight Self-Stigma Questionnaire (WSSQ) and type of region

The line chart (Figure 2) depicts the subjects' mean scores on the weight self-stigma questionnaire according to their region of residence. This demonstrates a statistically significant difference between different regions of Saudi Arabia with weight self-stigma being more prevalent in the Al-Baha region and least prevalent in Al-Madinah.

A linear regression using the forward method was run to predict weight-related self-stigma. BMI, psychological problems (DASS-21), region of residence, and age explained a significant amount of variance in weight-related self-stigma (F(4, 1583) = 59.225, p < 0.001, R2 = 0.13, R2 adjusted = 12.8). According to the regression coefficient, BMI (B = 3.638, 95% CI: 3.025, 4.251) significantly predicted weight-related self-stigma, corresponding to an increase in the WSSQ score of 0.278 points. Psychological problems (B = 0.138, 95% CI: 0.101, 0.175) corresponded to an increase of 0.173 in the WSSQ score. Region of residence (B = 0.276, 95% CI: 0.130, 0.422) and age (B = 0.215, 95% CI: 0.006, 0.424) corresponded to an increase of 0.087 and 0.048, respectively (Table 4).

**Table 4:** Linear regression analysis of factors independently predicting weight self-stigma CI, confidence interval; SE, standard error; BMI, body mass index; DASS-21, Depression, Anxiety, and Stress Scale 21.

Model	Unstandardized coefficients B	SE	Standardized coefficients (β)	t	p	95% CI	
						Upper	Lower
Constant	22.163	2.266	-	9.783	<0.001	17.719	26.607
BMI	3.638	0.313	0.278	11.642	<0.001	3.025	4.251
DASS-21 score	0.138	0.019	0.173	7.367	<0.001	0.101	0.175
Region of residence	0.276	0.075	0.087	3.699	<0.001	0.130	0.422
Age	0.215	0.106	0.048	2.020	0.044	0.006	0.424

## Discussion

This nationwide study examined associations between weight-related self-stigma and psychological distress in Saudi Arabian youths based on the work of a previous study [28]. Results demonstrated significant positive correlations between weight stigma, mental health issues, and BMI. Regression analysis indicated psychological distress and BMI were the strongest independent predictors of weight self-stigma. These findings align with prior research showing links between weight stigma and conditions like depression, anxiety, and stress [11,12,29-32].

Weight stigma can have well-established detrimental psychological impacts in overweight/obese populations. Some evidence suggests this relationship may be causal, with prospective studies linking perceived weight stigma to increased risk of stress and depression [33,34]. The correlations between weight stigma, depression, anxiety, and stress may stem from internalized weight bias and societal stereotyping/teasing faced by overweight individuals [30,35-37].

As shown in Figure 1, weight stigma correlates with distress, which can predict more stigma and disordered eating patterns [38,39]. Perceived weight discrimination doubles the risks of mood disorders and exercise avoidance, perpetuating stigma and obesity [40,41]. BMI and weight stigma are significantly associated, as higher BMIs increase vulnerability to stigma [35]. Though stigma-BMI links vary across studies [42,43], US data indicate people with higher BMIs report more stigma than minorities or mentally ill groups [44].

Our findings suggest that weight stigma is strongly tied to mental health issues like depression, anxiety, and stress among Saudi youth with overweight/obesity. Both biological and psychosocial factors are believed to contribute to this relationship [45,46]. Proposed biological mechanisms include inflammatory processes, hormonal dysregulation, and altered brain activity in areas regulating mood and emotions [45]. Psychosocial factors linking obesity and psychological distress encompass body dissatisfaction, poor self-esteem, social isolation, and experiences of weight stigma and bias [46]. Weight stigma can exacerbate distress directly through feelings of shame and embarrassment, as well as negatively impact mental health through maladaptive eating behaviors, reduced physical activity, and avoidance of health care [47,48]. This highlights the need for multifaceted clinical and public health efforts to address weight bias in this population. Counseling and therapy programs should aim to alleviate psychological distress related to weight stigma and teach adaptive coping strategies. Schools and communities can implement anti-bullying policies and stigma reduction campaigns. Healthcare professionals, including general practitioners, pediatricians, and other non-psychiatry specialists, should be trained in delivering compassionate, stigma-free care to patients with obesity. Additionally, parents and caregivers should be provided with education to increase awareness of weight stigma and its impacts on mental health in youth. Comprehensive policy and societal efforts are warranted to promote size acceptance and prevent weight discrimination against Saudi youths.

While this study provides initial evidence on weight stigma and mental health in Saudi Arabia, the cross-sectional design limits causal inferences. The reliance on self-report measures introduces the possibility of social desirability bias. The sample was predominantly female, limiting generalizability. Future longitudinal research with objective health measures, varied populations, and stigma interventions is needed to extend this work. Findings should be compared to studies in other Arab/Middle Eastern countries to identify cultural factors.

Despite being one of few regional studies examining weight self-stigma predictors using validated tools, this study has some limitations. The cross-sectional design cannot establish causality between weight stigma and associated factors. Additionally, the predominately female sample limits generalizability. However, as the first study in Saudi Arabia exploring weight self-stigma and psychological distress links, it can inform future efforts to mitigate weight stigma and its impacts. Further research should investigate causal relationships between weight stigma and predictors like quality of life using varied study designs. Our findings highlight the need for interventions to minimize weight stigma risk by addressing identified predictors. Weight stigma reduction should be a primary goal in obesity treatment and management programs. Regarding self-stigma differences, males reported higher self-stigma than females, aligning with past research using the Griffiths scale [49].

Our results also showed that respondents from the Al-Baha region had the highest enacted self-stigma scores, and those from Al-Madinah had the lowest scores. We believe this variation to be due to self-stigma being highly affected by social shame, thus regional awareness plays a significant role in stigmatization. However, this subject is poorly studied. Additional studies in this area are needed for a better understanding of the issue and to find the best solutions.

## Conclusions

The aim of this nationwide study was to investigate the association between weight-related self-stigma and psychological distress in Saudi Arabian youth. We examined relationships between weight self-stigma, depression, anxiety, stress, and BMI in a sample of 1624 adolescents and young adults. The results demonstrated significant positive correlations between weight self-stigma scores and the severity of depression, anxiety, and stress symptoms. Weight self-stigma also showed positive associations with BMI. These findings provide initial evidence of robust links between weight bias and adverse mental health outcomes among overweight/obese Saudi youth. This highlights weight stigma as a potential contributor to psychological issues in this population. The results can ultimately inform efforts to reduce weight bias and promote mental well-being through targeted clinical and public health initiatives.
